# A Rare Benign Tumor in a 14-Year-Old Girl

**DOI:** 10.1155/2018/1548283

**Published:** 2018-01-31

**Authors:** Meral Hassan Abualjadayel, Osama Y. Safdar, Maysaa Adnan Banjari, Sherif El Desoky, Ghadeer A. Mokhtar, Raed A. Azhar

**Affiliations:** ^1^King Abdulaziz University, Jeddah, Saudi Arabia; ^2^Center of Excellence in Pediatric Nephrology, King Abdulaziz University, Jeddah, Saudi Arabia; ^3^Pathology Department, King Abdulaziz University, Jeddah, Saudi Arabia; ^4^Urology Department, King Abdulaziz University, Jeddah, Saudi Arabia

## Abstract

**Background:**

Oncocytomas are the second most common benign renal neoplasm but, unfortunately, they are difficult to differentiate from renal cell carcinoma. Renal oncocytomas are rare and have mostly been reported in adults. To our knowledge, this is only the sixth pediatric reported case of renal oncocytoma worldwide.

**Case Presentation:**

A 14-year-old Yemeni girl with a recurrent history of urinary tract infections came to our clinic complaining of left flank pain with a frontal headache. Ultrasound showed a 3 cm, well-defined echogenic lesion with mild vascularity. This lesion increased in size on her subsequent follow-ups. Computed tomography showed no intralesional fat, vessels invasion, or enlarged lymph nodes. The patient underwent laparoscopic radical nephrectomy, and a pathology report confirmed the diagnosis of renal oncocytoma.

**Conclusion and Recommendations:**

We present the rare occurrence of renal oncocytoma in a pediatric patient and highlight the importance of considering oncocytomas in the diagnosis of a renal mass.

## 1. Background 

Most of the renal oncocytomas have been reported in the Western literature, and only a few cases have been detected in eastern populations. In the present study, we demonstrate the occurrence of renal oncocytoma in a pediatric patient in Saudi Arabia.

Renal oncocytoma was first described by Zippel in 1942 as a malignant entity [1]. However, in 1976, Klein and Valensi were able to demonstrate its benign characters based on their long series of 13 cases [2].

Renal oncocytoma is the second most common benign renal neoplasm after angiomyolipoma, accounting for 3–7% of all renal tumors [3]. Classical macroscopic characteristics of oncocytomas include a brown, well-demarcated lesion that has central stellate scar [4].

Unfortunately, most oncocytomas are difficult to differentiate from chromophobe renal cell carcinoma due to overlapping ultrastructural, immunohistochemical, and morphological characteristics [5]. Moreover, fine needle aspiration is often not diagnostic due to oncocytoma having similar cytological characteristics as renal cell carcinoma [6].

Development of renal oncocytoma in pediatric patients is rare. To our knowledge, this is only the sixth reported pediatric case of renal oncocytoma in a 14-year-old young girl.

## 2. Case Presentation

An 11-year-old Yemeni young girl presented with a history of recurrent urinary tract infections. Her past medical history was insignificant. Radiological investigations revealed that both kidneys are normal in size, position, and echogenicity, with bilateral renal fullness. Voiding cystourethrography was unremarkable, and follow-up ultrasound showed minimal fullness in the right renal pelvis.

At the age of 14 years old, she developed left flank pain along with a frontal headache. Ultrasound revealed a well-defined echogenic lesion measuring three centimeters at the lower pole of the left kidney, demonstrating mild vascularity, which was presumed to be angiomyolipoma. Further follow-up after three months revealed an increase in the size of the mass that measured around 3.22 × 3.8 cm with the same degree of vascularity (Figure 2).

A subsequent computed tomography (CT) scan showed an enhancing mass, measuring 4 × 5 cm, arising from the mid- and lower-left renal pelvis. No macroscopic intralesional fat was identified with no invasion of the renal vessels or enlarged lymph nodes (Figure 3).

By magnetic resonance imaging (MRI), we identified a solitary well-circumscribed left renal hilum mass that measured 5 × 3 × 5 cm. No vascular invasion was identified, and the mass showed avid enhancement in the arterial phase and minimal washout in the delay phase (Figure 4).

The patient underwent laparoscopic radical nephrectomy with an uneventful recovery. On regular follow-up, the patient was found to have persistent right kidney hydronephrosis, along with recurrent urinary tract infections, chronic kidney disease (stage three) with an estimated GFR of 58 ml/min/1.73 m^2^, and stage 2 hypertension.

**Figure 1:**
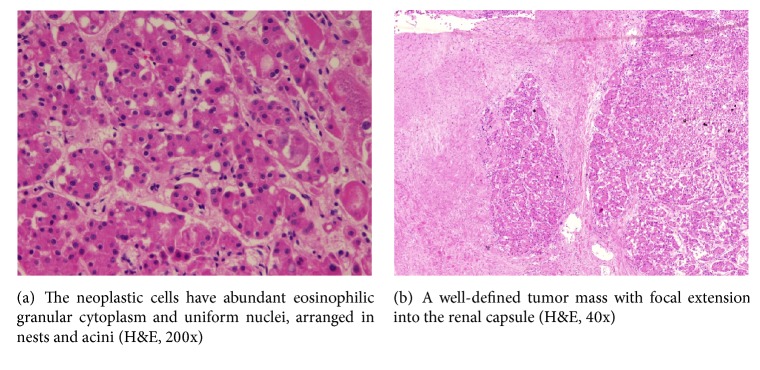
Pathology pictures.

**Figure 2:**
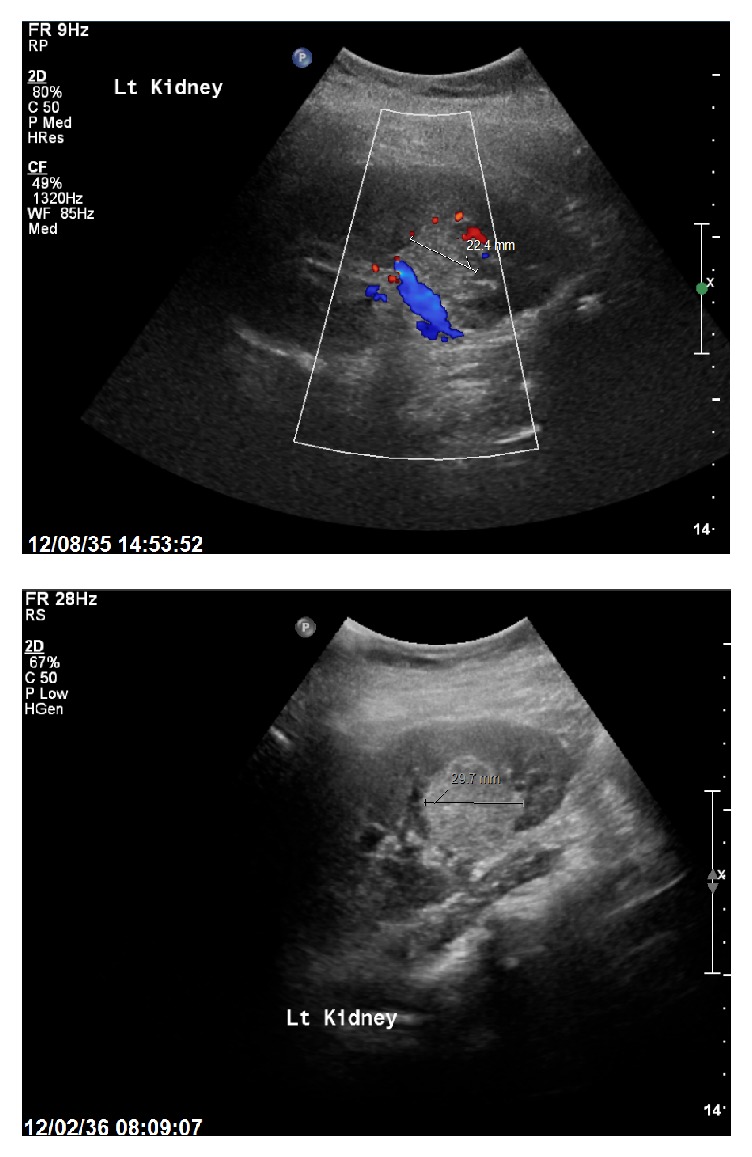
Ultrasound studies.

**Figure 3:**
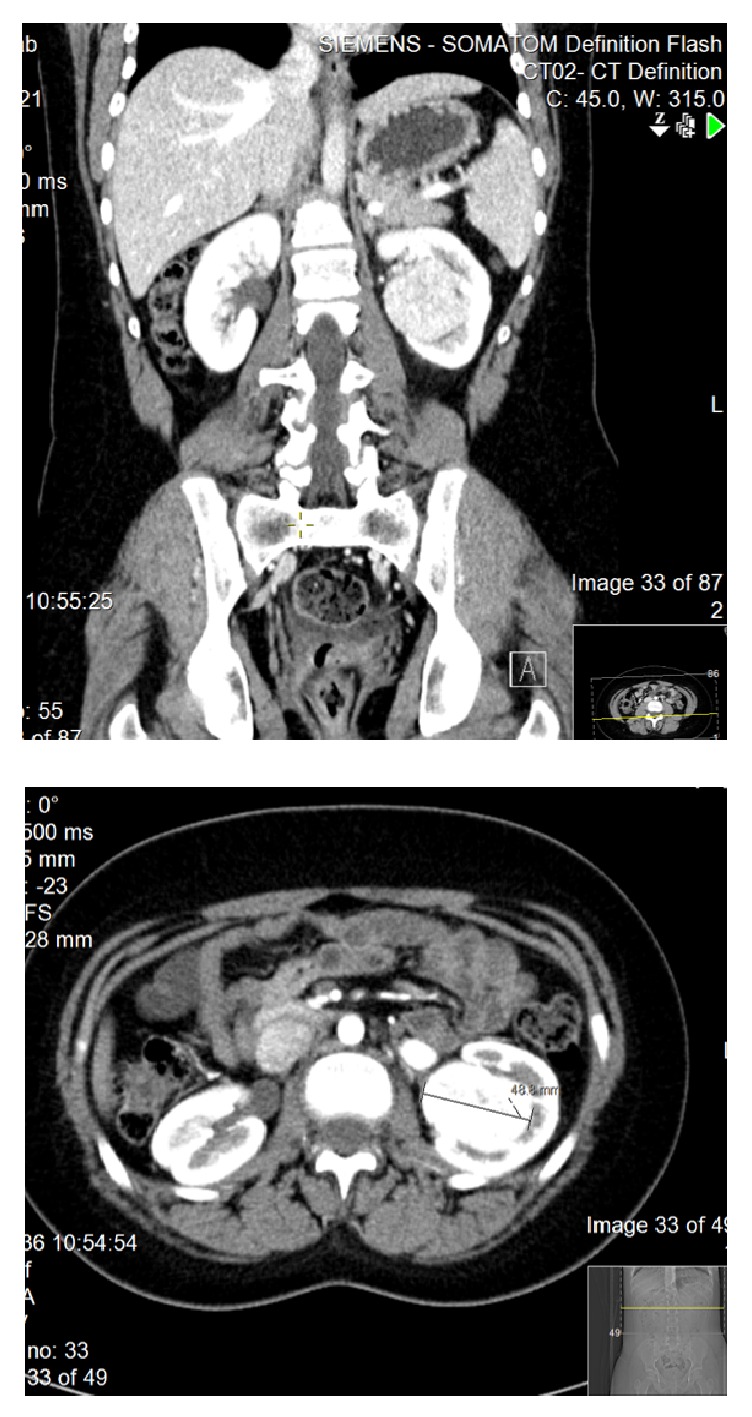
CT study.

**Figure 4:**
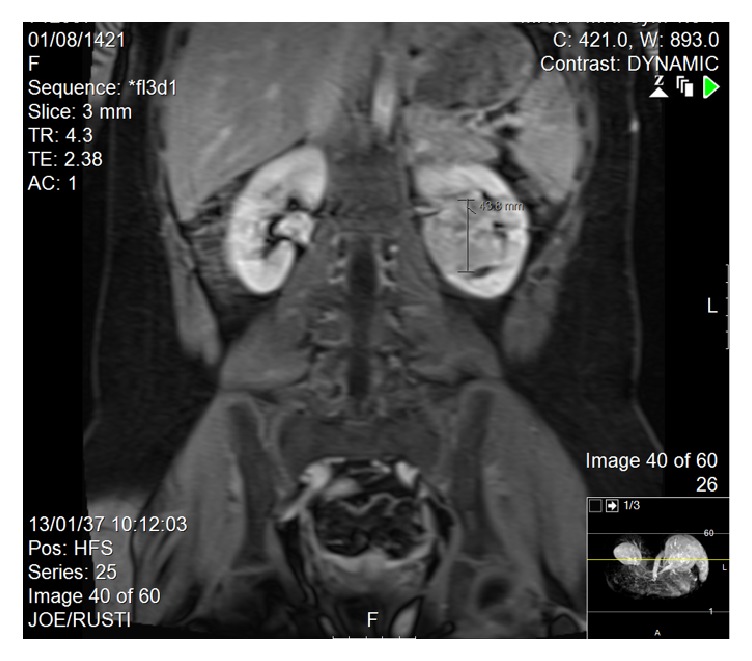
MRI study.

A pathology report revealed a well-defined tumor mass measuring 4 × 4 × 4 cm, located at the lower pole of the left kidney, brown, with foci of hemorrhage. No gross invasion of the renal capsule or perinephric fat was identified, and the adrenal glands were unremarkable (Figure 1).

Microscopic examination revealed a well-defined tumor composed of round to polygonal cells with abundant granular eosinophilic cytoplasm and regular, uniform nuclei, arranged in nests, acini, and tubules. Foci of hemorrhage were noted, and rare mitotic figures were identified. There was no evidence of necrosis. The nonneoplastic renal parenchyma shows only glomerular congestion and focal dilatation of the renal calyces. Hale colloidal iron histochemical staining was negative in the tumor cells. The neoplastic cells were also negative for vimentin and CK7 immunohistochemical stains.

## 3. Discussion

Renal oncocytoma is a rare benign tumor that has unexpectedly occurred in our young patient regardless of the known mean age of this tumor, which strongly influenced the quality of her life.

Oncocytoma usually occurs during the seventh decade of life, varying from 20 to 86 years of age with a predominance in males [7]. Most oncocytomas are single and unilateral, although, occasionally, they can be bilateral and multifocal [8]. The size of these tumors typically varies from 0.6 to 14 cm [9].

Most oncocytomas are diagnosed incidentally when the patients are assessed for nonurological complaints. However, symptomatic patients usually manifest with gross hematuria, flank pain, or a palpable mass [10]. Even though renal oncocytoma is known to have excellent long-term outcomes [11], the diagnosis is made after surgical removal of the tumor because of lack of specific clinical features and imaging findings [12]. Patients with renal oncocytomas need to be monitored closely for any evidence of coexisting renal cell carcinoma or rapid growth in size [10].

In renal oncocytoma patients, CT scans reveal a homogenous well-circumscribed solid mass with a central stellate scar [13]. However, this is only found in 33% of cases [14]. In our case; no central scar was visible by CT.

Furthermore, MRI of renal oncocytoma typically produces homogenous images with low density in T1 weighted sequences, which becomes hyperintense in T2 weighted images [15].

There have been some genetic abnormalities associated with renal oncocytomas, such as loss of chromosome Y or translocations in the 11q13 and loss of heterozygosity on chromosome 14q [10, 12]. However, genetic testing was not performed in this study.

Rarely, oncocytoma can occur as a part of hybrid oncocytic chromophobe tumors (HOCT), which are a subtype of chromophobe renal cell carcinoma (chRCC) that consist of chRCC and renal oncocytoma. This unique presentation has only been reported in two pediatric cases [16, 17]. Furthermore, researchers have shown that primary clear cell carcinoma can coexist with oncocytoma in the same or contralateral kidney [18].

Renal oncocytoma is more prevalent in adults than in children. A literature review revealed that, among the five documented pediatric cases, the youngest age was 10 years, and the eldest was 17 years; most pediatric patients complained of flank pain as a presenting symptom, which is similar to our case. Male to female predominance was 1 : 1 and no cases had any genetic association evident at the time of publication (Table 1) [19].

## 4. Conclusion

In this case, we present the rare occurrence of renal oncocytoma in a pediatric patient and the importance of considering it as part of the differential diagnosis of a renal mass.

## Figures and Tables

**Table 1:** Review of case reports of the occurrence of renal oncocytoma in pediatric patients.

Sex	Age	Presentation	Treatment	Genetic association
Male (20)	13 years old	Left flank pain	Partial nephrectomy	None
Male (21)	17 years old	Viral illness, abdominal mass	Nephrectomy	None
Male (22)	10 years old	Recurrent abdominal pain, malaise, anorexia, macroscopic hematuria and dysuria	Nephrectomy	None
Female (23)	12 years old	Left sided abdominal fullness	Nephrectomy	None
Female (19)	12 years old	Right flank pain	Nephrectomy	None
Female (this case)	14 years old	Left flank pain	Nephrectomy	None
